# Exclusive Breastfeeding Predicts Higher Hearing-Language Development in Girls of Preschool Age

**DOI:** 10.3390/nu12082320

**Published:** 2020-08-02

**Authors:** Maria Angela Guzzardi, Federico Granziera, Elena Sanguinetti, Francesca Ditaranto, Filippo Muratori, Patricia Iozzo

**Affiliations:** 1 Institute of Clinical Physiology, National Research Council (CNR), 56124 Pisa, Italy; m.guzzardi@ifc.cnr.it (M.A.G.); granziera.federico@gmail.com (F.G.); elenamariposa@libero.it (E.S.); 2 IRCCS Fondazione Stella Maris, Calambrone, 56128 Pisa, Italy; francescaditaranto@hotmail.com (F.D.); fmuratori@fsm.unipi.it (F.M.)

**Keywords:** breastfeeding, cognitive development, maternal obesity

## Abstract

Cognitive disorders are increasing in prevalence. Nutritional or metabolic stressors during early life, and female sex, are predisposing conditions towards the development of cognitive diseases, including Alzheimer’s disease. Though there is evidence that breastfeeding may play a beneficial role in children’s neurocognitive development, the literature remains controversial. In this study we aimed at assessing the association between exclusive breastfeeding and children’s cognitive development from six months to five years of age, addressing sex differences. In 80 mother-child pairs from the Pisa birth cohort (PISAC), we measured cognitive development in groups of children of 6, 12, 18, 24, 36, and 60 months by Griffiths Mental Development Scales, parents’ intelligence quotient (IQ) by Raven’s progressive matrices, and maternal and infants’ anthropometric parameters. We found that exclusive breastfeeding was associated with higher hearing-language development in five years old girls, independent of maternal IQ, age and BMI (body mass index). Exclusive breastfeeding in the first three months of life seemed sufficient to establish this positive relationship. In conclusion, our data indicate that exclusive breastfeeding is a positive predictor of cognitive development in preschool-age girls, paving the way for the implementation of sex-specific cognitive disease risk detection and prevention strategies from early life. Further studies are warranted to explore causality and longer term effects.

## 1. Introduction

Exposure to metabolic, stress and nutritional factors during early life can shape neuronal development and brain function in later life. Among these factors, early feeding, in particular lactation, has raised research interest because the developing brain is greatly susceptible to postnatal nutritional deficits [1,2].

Breastfeeding was shown to be protective against later development of obesity and metabolic diseases in offspring when compared to formula feeding [3,4,5]. Conversely, the effect of breastfeeding on cognitive development is still controversial. In fact, several studies report positive associations between breastfeeding duration and exclusivity and cognitive and school achievements during childhood [6,7,8,9,10,11,12] and adulthood [13], whereas no association was reported in other studies [14,15,16].

Several methodological issues can contribute to explain discrepancies among studies. One source of heterogeneity is represented by the different definitions of breastfeeding. Breastfeeding can be measured by duration, exclusivity or volume of breast milk consumed. The duration criterion carries an intrinsic confounder due to the frequent addition of formula in variable proportions (and different brands), whereas the objective measurement of breast milk consumed is technically very challenging.

Other methodological issues include the diverse children’s ages and the methods used for assessing children’s cognition, as well as the confounders that need to be considered [6]. Previous studies have demonstrated that maternal intelligence quotient is an important confounder in the association between breastfeeding and cognitive performance [17], and that maternal obesity is a negative predictor of offspring cognitive development [18,19,20]. In addition, increasing parental age, especially maternal age, seems associated with language, communication and cognitive abilities with a reverse J-shaped relationship [21,22]. Though these maternal factors should be taken into account when studying the association between breastfeeding and cognitive performance, the correction for these variables is not reported in most studies.

In the present study, we aimed at assessing the relationship between breastfeeding and cognitive development from the age of six months to five years in a subset of the Pisa birth cohort (PISAC), including families that were recruited during pregnancy and followed-up to the children’s age of five years.

To overcome the above biases, we stratified our population in two groups, separating children who had received exclusively breast milk from those who had received any combination of breast and formula milk before weaning. Moreover, we measured cognitive scores of both parents and children objectively, by standard tests, and we recorded mothers’ and offsprings’ body sizes as potential confounders. We focused on preschool age to avoid e.g., exposure to the school environment and other social stimuli, which make it more difficult to dissect early determinants of cognitive development. Finally, we accounted for the epidemiological observation that women have a higher risk to develop cognitive disease, including Alzheimer’s disease [23]. Sex influences the levels of hormones and hormone-receptors in all organs, including the brain. The prenatal hormone environment contributes to sex-specific neurodevelopmental and behavioral outcomes in the offspring [24]. After birth, human milk is a further source of nutrients and hormones, and sex-specific differences in brain response could influence brain development [25]. Therefore, we analyzed the association between breastfeeding and cognitive outcomes in girls and boys separately, to support a better identification of subjects who may benefit from attention and prevention since early-life.

## 2. Material and Methods

### 2.1. Study Population

The study was conducted in the Pisa birth cohort (PISAC). The cohort includes 90 families, i.e., mothers, fathers, and infants born 2011–2014, 42/48 females/males, followed up from birth. Families were recruited at the beginning of pregnancy visits or at delivery (41/49) through hospital pregnancy visits in the area of Massa, Tuscany, Italy, to investigate the effects of maternal obesity during pregnancy on offspring cardiometabolic and cognitive health. The cohort is intended to represent the general population, and therefore inclusion criteria are broad, namely (1) mothers within the first trimester of pregnancy at the first visit or at delivery; (2) any parents’ age; (3) any BMI; (4) willingness of mothers and fathers to participate and to actively collect questionnaires and samples; (5) capacity of mothers and fathers to understand the study and its implications; (6) signature of the informed consent by mothers and fathers and (7) absence of major diseases (mothers and fathers) and perinatal complications. Exclusion criteria are (1) history of major diseases in the mother and in the father (kidney failure, liver failure, cardiac failure, major lung disease, autoimmune disease, cancer, psychiatric illness, also including anorexia-bulimia nervosa and substance abuse); (2) major health complications during the perinatal period and (3) failure to understand study implications, comply with the study schedule or sign the consent form. Follow-up visits were carried out from birth to the children’s age of five years and included measurement of anthropometric parameters, blood pressure, complete echocardiographic evaluation, cognitive assessment and collection of nutritional questionnaires and biological samples (e.g., stools and urine). Families were given the option to participate in all or only part of the assessments and measurements included at each age-point. In addition, we prioritized different health aspects at different age points, namely cardiac health in the first two years and cognitive health in preschool five-year old children. Therefore, the sample size varied across age point and measurements. At age five years, all families were called for a new follow-up round, only six decided to drop-out, while the others participated in all or some measurements, or asked to be considered for future follow-up rounds. The cognitive follow-up at five years included 56 mother-offspring dyads that were homogenous with the enrolled population in terms of mothers’ age (34.0 ± 0.5 vs. 33.3 ± 0.5 years) and fathers’ age (36.6 ± 0.6 vs. 36.2 ± 0.5 years). Distribution of mothers’ or fathers’ job categories was not related to the type of feeding categories (i.e., exclusively and nonexclusively breastfeed), nor to adherence to any of the cognitive follow-up visits. Body sizes were collected at the beginning and during pregnancy in mothers, and in mothers and infants at multiple time points after delivery. In women, and in 36 and 60 months-old children, body weight (in kg to the nearest 0.1 kg) and length (in cm to the nearest 0.5 cm) were measured by weight scale and stadiometer, with participants wearing light clothes and standing straight without shoes and with heels close together. In infants up to age two years, weight was measured by using a hospital-grade pediatric weighting scale (in kg to the nearest 0.01 kg) removing clothes, shoes and diaper from the infants, and recumbent length (in cm to the nearest 0.1 cm) was recorded. Additional body size measurements were collected from pediatric records. Information on feeding was collected at three and six months, children’s and parents’ cognitive scores were measured starting from the infants’ age of six months, as reported in the following paragraph.

The study was conducted in accordance with the Declaration of Helsinki and was approved by the Ethics Committee of Massa and Carrara, and latest amendments by the Ethical Committee of the Area Vasta Nord-Ovest (CEAVNO), Pisa, Italy (study identification code 394, approval decrees n. 75 and 71512). Parents gave their written informed consent before inclusion.

### 2.2. Definition of Feeding Type

Information on early feeding were collected via parents’ interview during follow-up visits or by phone at three and six months of age, and they were used to stratify children in two groups, i.e., one receiving exclusive and one receiving nonexclusive breastfeeding before weaning. Infants receiving only maternal milk (i.e., no addition of formula milk) before the introduction of any complementary food were defined as exclusively breastfed. Conversely, the nonexclusive breastfeeding category included infants who received only formula milk or a combination of breast and formula milk in any proportion. Eighty of the 90 families provided information on early feeding practices and were included in this work. These 80 families were homogenous with the enrolled population in terms mothers’ age (33.4 ± 0.5 vs. 33.3 ± 0.5 years) and fathers’ age (36.4 ± 0.5 vs. 36.2 ± 0.5 years).

### 2.3. Measurement of Children’s and Parents’ Cognitive Scores

Children’s cognitive development was assessed by a trained childhood psychologist by using the Griffiths Mental Development Scales (GMDS) in subgroups of children at the age of 6, 12, 18, 24 and 36 months (*N* = 24, 26, 26, 23, 27). At the age of five years, cognitive development was measured again in a larger subset (*N* = 56). The GDMS measures the rate of development of infants and young children in six domains, i.e., locomotor, personal-social, hearing and language, eye and hand coordination, performance and practical reasoning domains (the latter from age 36 months). Mothers’ and fathers’ intelligence quotients (IQ) were measured by the Raven’s progressive matrices (*N* = 58 and 36, respectively).

### 2.4. Statistical Analysis

SPSS for Mac (version 22, Chicago, IL, USA) was used for statistical analyses. Variables distribution was tested by the Shapiro-Wilk test. A two-tailed *t*-test was used for group comparisons, and one-way ANCOVA (analysis of covariance) was used to incorporate covariates (i.e., mother’s and father’s age, mother’s BMI and IQ, children’s sex and weight gain). To assess the sex-specific relationship between breastfeeding and cognitive development, we repeated each analysis in girls and boys separately. The Chi-Square test was applied to examine relationships between categorical variables. These tests were selected to capture group differences in cognitive outcomes at each separate age point, maximizing power. Considering that not all children had cognitive measures at all-time points, changes over time by repeated measure testing was not performed to avoid sample size reduction. Results are presented as mean ± sem, and *p*-values ≤ 0.05 were regarded as statistically significant.

## 3. Results

### 3.1. Characteristics of Exclusively and Nonexclusively Breastfed Children

In our population, 50% of children were exclusively breastfed. General characteristics of the stratified population are reported in Table 1. Among nonexclusively breastfed children, 65% (*n* = 26) received both maternal and formula milk between birth and weaning (mean age 5.4 ± 0.1 months in the whole population). Exclusive formula milk feeding from birth to weaning occurred in 35% (*n* = 14), while 27% (*n* = 11) of the infants were started on exclusive formula feeding at the age of three months. The prevalence of maternal gestational diabetes, as well as children’s sex distribution and delivery mode were not different between the two groups of children.

### 3.2. Relationship between Exclusive Breastfeeding and Children’s Cognitive Development

Cognitive scores measured in six different domains were in the normal range [26], both in males and in females. However, females had higher scores compared to males at 36 months in the personal-social domain (103.8 ± 2.7 vs. 97.9 ± 1.5 *p* = 0.046) and at five years in the eye and hand coordination domain (102.4 ± 2.7 vs. 97.3 ± 1.7 *p* = 0.040) and in the performance (114.0 ± 1.3 vs. 110.5 ± 1.3 *p* = 0.025) domain.

**Figure 1:**
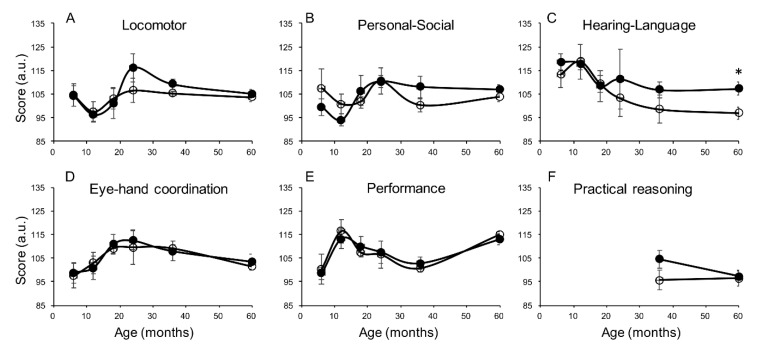
Cognitive development measured in the six cognitive domains (**A**–**F**) in exclusively breastfed (closed circles) and nonexclusively breastfed (open circles) girls at different ages (6, 12, 18, 24, 36, 60 months). *N* = 9, 10, 14, 10, 13, 25 at progressive ages. Practical reasoning skills are measured from the age of 36 months. Data are represented as mean ± sem. * *p* ≤ 0.05.

**Figure 2:**
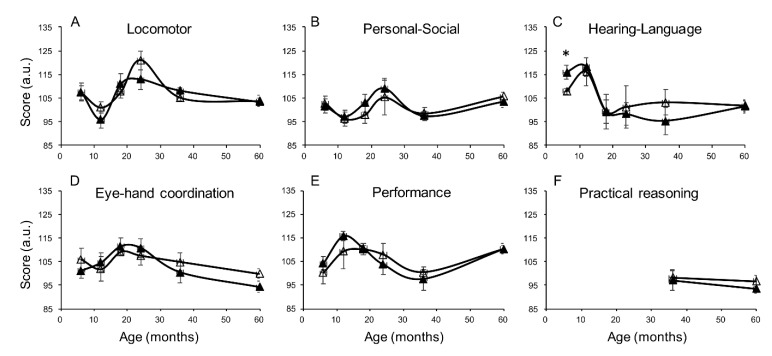
Cognitive development measured in the six cognitive domains (**A**–**F**) in exclusively breastfed (closed triangles) and nonexclusively breastfed (open triangles) boys at different ages (6, 12, 18, 24, 36, 60 months). *N* = 15, 16, 12, 13, 14, 30 at progressive ages. Practical reasoning skills are measured from the age of 36 months. Data are represented as mean ± sem. * *p* ≤ 0.05.

In the whole population, accounting for sex as a covariate, exclusive breastfeeding was associated with higher scores in hearing and language at six months (*p* = 0.049) and locomotor development at 36 months (*p* = 0.036). In separate sex group analyses (Figure 1 and Figure 2), girls receiving exclusive breastfeeding had significantly higher hearing and language scores at the age of five years compared to nonexclusively breastfed girls (Figure 1C, *p* = 0.014, Table 2). This finding was already visible at 36 and at 24 months, falling short of significance due to the smaller sample size. Similar trends, showing higher scores in exclusively compared to nonexclusively breastfed girls were observed in the locomotor domain (Figure 1A) and in the personal-social domain (Figure 1B). In males, exclusive breastfeeding was associated with a higher score in the hearing-language scale at six months (Figure 2C, *p* = 0.021), with no significant difference in other scales and other ages.

### 3.3. Impact of Parental Intelligence and Age on the Relationship between Exclusive Breastfeeding and Children’s Cognitive Development

In our population, the maternal IQ was directly associated with the hearing and language scores at 18 months (r = 0.416, *p* = 0.043) and at 36 months (r = 0.542, *p* = 0.007), with practical reasoning at 36 (r = 0.663, *p* = 0.001) and at 60 months (r = 0.366, *p* = 0.009), and with personal-social scores at 36 months (r = 0.599, *p* = 0.003), while being negatively correlated with the infants’ personal-social (r = −0.484, *p* = 0.017) and performance (r = −0.488, *p* = 0.015) scores at six months. Fathers’ IQ was directly associated with offspring performance at 36 months (r = 0.583, *p* = 0.006) only. However, the IQ was measured in a smaller subset of fathers, and was not used in covariate analysis to avoid sample size and power reduction.

Parental age was also related to children’s cognitive development. In particular, maternal age was directly associated with the performance score at 60 months (r = 0.282, *p* = 0.025) and negatively with the locomotor (r = −0.433, *p* = 0.034) and performance (r = −0.427, *p* = 0.038) scores at six months. Fathers’ age was negatively correlated with the locomotor score at 36 months (r = −0.399, *p* = 0.039).

In the whole children’s population, the adjustment for maternal IQ and/or parents’ age abolished the association between exclusive breastfeeding and locomotor scores at 36 months, but not the association between exclusive breastfeeding and hearing and language scores at six months (*p* = 0.046), in which exclusive breastfeeding was able to explain 20% of the difference between children’s groups. Likewise, in sex-specific analyses, the adjustment for maternal IQ and parents’ age did not abolish the difference observed in hearing and language scores at five years in girls (*p* = 0.034 and 0.043, respectively), in which exclusive breastfeeding was able to explain 22% of the difference between groups.

Similar results in both uncorrected and corrected analyses were obtained considering exclusive breastfeeding within the first three months only (instead of six months), suggesting that the first trimester of life may be sufficient to predict the development of hearing and language skills in girls.

### 3.4. Impact of Maternal and Children’s Body Weight on the Relationship between Exclusive Breastfeeding and Children’s Cognitive Development

Exclusive breastfeeding was significantly less common in women with a high than low BMI in our maternal population (Table 1), independent of offspring sex (*p*-values between 0.012–0.040 in sex-specific analyses). However, the maternal BMI did not correlate with cognitive outcomes in the offspring at any time point. Instead, a lower weight gain in infants between three and six months of age was associated with exclusive breastfeeding (*p* = 0.006 and 0.042 in males and females, respectively), and negatively correlated with hearing and language scores at five years in the whole population (r = −0.310, *p* = 0.025), showing a trend in females (r = −0.376, *p* = 0.064).

Children’s body weight, length (not reported), BMI and cranial circumferences at birth and at 3, 6, 12, 18, 24, 36 and 60 months of age were similar between the two groups and not related to cognitive outcomes.

In the fully adjusted analysis, we found that exclusive breastfeeding was still related to higher hearing and language scores in five years old girls, independent of maternal pregravidic and gravidic BMI (*p* = 0.033) and of children’s weight gain in the first six months of life (*p* = 0.04), accounting for 20% of the group difference (Table 2).

## 4. Discussion

The main finding of the present study is that exclusive breastfeeding before weaning age (i.e., in the first six months of life) shows a strong and positive association with hearing and language scores in girls reaching the age of five years. Moreover, exclusive breastfeeding in the first three months of life seems sufficient to observe this effect.

We also showed that exclusive breastfeeding is associated with lower maternal BMI and lower infants’ weight gain between three and six months of age. A higher weight gain in this time window negatively predicts hearing and language scores at five years of age. Moreover, parents’ IQ and age predict offspring cognitive development. However, none of these factors affected the main finding.

Several authors have explored the role of early feeding practices on children’s cognitive development, producing sparse and controversial results. Some studies found positive associations between breastfeeding duration and exclusivity and cognitive and school achievements during childhood [6,7,8,9] and adulthood [13]. In a medium-large population of approximately 500 children, breastfeeding was positively related to neurological development at nine years [27]. There are also few studies in which no positive relationship between breastfeeding and children’s cognitive development was reported [14,15,16]. It is important to underline that most of above studies cover both prescholar and scholar age [9,13,14] or were conducted in older scholar age children only [8,16]. We focused on preschool age in order to avoid the exposure to school educational and social stimuli and better dissect the contribution of early determinants on cognitive development. Targeting the same age, our results are in agreement with, and extend to a Mediterranean population the finding reported in a cohort of 10,700 four-year old American children, in which Jenkins et al. [10] showed a small but significant association between exclusive breastfeeding and reading abilities and math scores. Similarly, a study in more than 1300 American mothers and children demonstrated a positive relationship between exclusive breastfeeding and both verbal and nonverbal intelligence at the age of three years [12].

Another source of discordant results is given by the heterogeneous definitions of breastfeeding and the lack of adjustment for important confounders. For example, in an old study, no difference in phonologic development at the age of three to four years was observed between children who had been breastfed or bottle fed [28]. A large American prospective study involving more than 5000 children born in the eighties concluded that being breastfed does not associate with cognitive development [14]. However, in these reports a different stratification criterion was used, as children who had never received breast milk were compared to all the others. Therefore, breastfeeding in these studies included a proportion of bottle feeding in some infants, not being exclusive. Our intent was to dissect the specific predictive value of breastfeeding on cognitive development. Therefore, we separated children who had received breast milk exclusively from all the others, in order to obtain a group with homogeneous breastfeeding behavior and avoid any mixed effect related to variable breast-to-formula milk ratios.

Several authors [14,17] suggest that the positive correlation between breastfeeding and children’s cognition may be mediated by the confounding action of maternal intelligence, which is not objectively measured and controlled for in a majority of studies. The important role of maternal IQ and family educational level is underscored also by Gale at al [29]. who showed that controlling for these parameters greatly attenuated the difference in full-scale and verbal scores observed between four-year old children fed with breast versus formula milk. One strength of our study was to control for parental IQ, showing that the positive association between exclusive breastfeeding and hearing and language skills in five years old girls was not abolished after adjusting for maternal IQ, as objectively measured by Raven’s matrices.

The duration of breastfeeding, besides its exclusivity, could also be relevant to predict children’s cognitive development. Stratification of our population based on exclusive breastfeeding at three rather than six months, resulted in similar associations between breastfeeding and cognitive outcomes, which suggests that the first three months could be sufficient to positively predict hearing and language development. In agreement with our results, exclusive breastfeeding even for only three months was related to higher intelligence quotient in more than 450 Polish children of prescholar age [30]. In the large American population reported above [10], longer periods of exclusive breastfeeding produced slight increases in effect size when considering reading but not math skills, but a clear dosage effect in the relationship linking exclusive breastfeeding and cognitive outcomes was not found. Conversely, Angelsen et al [31]. showed that shorter duration of breastfeeding, categorized as shorter than three months, between three to six months, or longer than six months, was associated with lower scores of mental development at one and five years of age in 345 Scandinavian children. However, in the latter study, exclusivity of breastfeeding was not taken into account. Current recommendations from international health organizations (e.g., WHO) indicate six months as the optimal exclusive breastfeeding duration for the health and well-being of both child and mother. Our data support and extend this recommendation by suggesting that, within the limits of selected cognitive outcomes in girls, a shorter exclusive breastfeeding duration, likely fitting a broad population, is already a significant and positive predictor.

Our results extend previous observations in preschool children since we explored multiple ages within the first five years of life. One additional novel observation is the sex difference emerging from our study. In fact, we showed a positive independent correlation between exclusive breastfeeding and cognitive outcomes in girls but not in boys. The analysis of specific differences between males and females is mostly neglected in the literature addressing early nutrition. It is now established that neurodevelopment during fetal and subsequent life is influenced by hormones [32,33], and that maternal milk is a source of nutrients and hormones. However, not much is known on the sex-specific hormones and brain interaction at this stage. Studies on primates have shown sex-specific effects of early nutrition on offspring outcome, e.g., females would show higher susceptibility to the effect of prenatal nutrition than males [34], whereas high cortisol in the milk has been related to more nervousness and less confident temperament in male, rather than in female, offspring [35]. Recently, sexual dimorphism in human milk hormonal composition has been shown, in particular in insulin-like growth factor-1 (IGF-1) and adiponectin concentration [25]. Both hormones are important for brain health. IGF-1 has been implicated in neurodevelopment, both prenatally and in the early post-natal period, and in plasticity and remodeling throughout life [36], and adiponectin seems to play a role in the onset of cognitive decline and in Alzheimer’s disease [37]. Finally, most chronic diseases show sexual dimorphism, and in the field of cognitive decline, including Alzheimer’s disease, women seem to be at greater risk than men, especially with aging [23]. Demonstration of a different sensitivity to exclusive breastfeeding in girls than boys may serve to select subjects who may benefit from nutritional attention and prevention since early-life.

Obesity, either in the mother during pregnancy or in the children, is considered a negative predictor of cognitive outcomes [18,19,20,38,39], and should be taken into account when exploring the relationship between early feeding and cognitive development.

In our study, exclusive breastfeeding was significantly less common in women with a high than low BMI, in line with the established inverse association between maternal obesity and low rates of initiation, duration and exclusivity of breastfeeding, as published by Li et al. [40] in a population of more than 124,000 mother-infant pairs, in line with other reports [41]. Conversely, no differences were detected in body sizes, including BMI, between exclusively and nonexclusively breastfed children, consistent with results from the above American population [10]. However, exclusively breastfed children underwent lower weight gain than nonexclusively breastfed children between three and six months of age. This is consistent with the lower weight gain velocity within the first 12 months of life observed by Azad et al. [3] in breastfed children of the the Canadian Healthy Infant Longitudinal Development (CHILD) study involving over 2500 mother-infant dyads. Higher weight-gain in infancy is predictive of later overweight or obesity, which has been related to poorer cognitive development and scholar performance [38,39,42,43]. Consistently, in our population, higher children’s BMI at five years was associated with lower practical reasoning score at the same age, and higher weight-gain between three to six months was associated with a worse hearing and language development at five years.

Of note, in our study the positive correlation between exclusive breastfeeding and hearing and language scores in five-year old girls were independent of the above negative predictors, i.e., maternal pregravid and gravid BMI, and children’s weight gain.

We acknowledge that our study has limitations. The small sample size, and the fact that most children could not undergo all repeated measures, might have prevented our analyses from finding more significant differences among cognitive domains before the age of five years. In fact, relationships were stronger in the most numerous group of *n* = 56 children of five years. In addition, multiple correlations bear the risk of chance findings, though our main observation was robust and remained fully significant after adjusting for confounders. The second limitation, which is a frequent criticism in breastfeeding targeted research, is the intrinsic bias of feeding groups, since infants are not randomly allocated to groups, and parents’ and family characteristics may be responsible for both feeding choices and cognitive outcomes. To mitigate this risk, we controlled for maternal features associated with cognitive scores and/or feeding practices, i.e., BMI and IQ. The strengths of our study are that both parents’ and children’s cognitive scores were objectively measured and not estimated from proxy variables such as school or job attainment or questionnaires, and that cognition was examined at different ages in preschool children.

## 5. Conclusions

In conclusion, we found that exclusive breastfeeding predicts higher hearing and language outcomes in five-year old girls, independent of parents’ age, maternal weight and IQ, or children’s weight and weight gain. Knowing that cognitive decline is frequent in women, our finding of a sex-selective association offers an opportunity to identify individuals at risk in a very early stage of life, requiring nutritional attention. Considering that formula feeding is not a choice of families in many cases, but depends on an insufficient production of breast-milk, or the return to work after the first postnatal months, it was important to show that three months of breast-feeding already predicted the beneficial outcome. Caution should be used in the interpretation of data, which are mainly correlative and do not imply causality or long term benefits, representing the challenges of future studies.

## Figures and Tables

**Table 1:** Characteristics of exclusively and nonexclusively breastfed children.

Variable	*N*	Exclusive Breastfeeding	Nonexclusive Breastfeeding	*p*-Value
Mixed/formula feeding (*N*)	80		26/14	<0.0005
Sex (Females/Males)	80	16/24	24/16	n.s.
Delivery type (Vaginal/C-section)	80	24/16	28/12	n.s.
Gestational diabetes (N)	80	12	10	n.s.
Gestational age (days)	80	277 ± 1	277 ± 2	n.s.
Weaning age (months)	77	5.6 ± 0.1	5.3 ± 0.1	n.s.
Mother age (years)	80	33.2 ± 0.7	33.7 ± 0.7	n.s.
Father age (years)	80	35.3 ± 0.7	37.5 ± 0.8	0.04
Mother pregravidic BMI (kg/m^2^)	80	23.1 ± 06	26.8 ± 1.0	0.002
Mother gravidic BMI (kg/m^2^)	75	28.4 ± 0.6	31.6 ± 1.1	0.016
Mother IQ (RPM score)	58	112.0 ± 1.9	113.1 ± 2.4	n.s.
Father IQ (RPM score)	36	114.2 ± 2.3	117.9 ± 2.9	n.s.
Birth weight (kg)	80	3.36 ± 0.05	3.33 ± 0.07	n.s.
Birth BMI (kg/m^2^)	80	13.5 ± 0.2	13.3 ± 0.2	n.s.
Birth PI (kg/m^3^)	80	27.0 ± 0.35	26.65 ± 0.41	n.s.
Weight at 3 months (kg)	73	6.09 ± 0.14	5.94 ± 0.12	n.s.
Weight at 6 months (kg)	72	7.64 ± 0.15	7.88 ± 0.15	n.s.
Weight at 12 months (kg)	71	10.03 ± 0.23	10.13 ± 0.20	n.s.
Weight at 18 months (kg)	60	11.24 ± 0.28	11.46 ± 0.22	n.s.
Weight at 24 months (kg)	53	12.73 ± 0.40	12.91 ± 0.36	n.s.
Weight at 36 months (kg)	54	15.14 ± 050	15.44 ± 0.38	n.s.
Weight at 60 months (kg)	66	20.18 ± 0.67	20.77 ± 0.58	n.s.
BMI at 3 months (kg/m^2^)	72	16.5 ± 0.3	16.3 ± 0.2	n.s.
BMI at 6 months (kg/m^2^)	72	16.5 ± 0.5	17.2 ± 0.3	n.s.
BMI at 12 months (kg/m^2^)	69	17.2 ± 0.3	17.1 ± 0.2	n.s.
BMI at 18 months (kg/m^2^)	57	16.6 ± 0.3	16.7 ± 0.3	n.s.
BMI at 24 months (kg/m^2^)	51	16.6 ± 0.4	16.5 ± 0.3	n.s.
BMI at 36 months (kg/m^2^)	52	16.5 ± 0.4	16.6 ± 0.3	n.s.
BMI at 60 months (kg/m^2^)	66	16.8 ± 0.5	17.1 ± 0.4	n.s.
Weight gain 0–3 months (kg)	73	2.71 ± 0.11	2.61 ± 0.10	n.s.
Weight gain 3–6 months (kg)	69	1.57 ± 0.07	1.93 ± 0.10	0.003
Weight gain 0–6 months (kg)	72	4.28 ± 0.12	4.54 ± 0.13	n.s.
Weight gain 0–12 months (kg)	71	6.67±0.20	6.81 ± 0.19	n.s.
Weight gain 0–60 months (kg)	66	16.84 ± 0.65	17.41 ± 0.56	n.s.
Weight gain 12–24 months (kg)	51	2.84 ± 0.18	2.80 ± 0.30	n.s.
Weight gain 24–36 months (kg)	46	2.07 ± 0.22	2.52 ± 0.21	n.s.
Weight gain 36–60 months (kg)	51	5.26 ± 0.43	5.48 ± 0.46	n.s.
Cranial circ. at 3 months (cm)	48	40.1 ± 0.2	40.2 ± 0.3	n.s.
Cranial circ. at 6 months (cm)	49	42.7 ± 0.2	43.0 ± 0.3	n.s.
Cranial circ. at 12 months (cm)	53	46.2 ± 0.2	45.9 ± 0.3	n.s.
Cranial circ. at 18 months (cm)	24	47.5 ± 0.4	47.5 ± 0.4	n.s.
Cranial circ. at 24 months (cm)	13	49.2 ± 0.6	49.5 ± 0.5	n.s.
Cranial circ. at 36 months (cm)	24	50.4 ± 0.5	50.9 ± 0.4	n.s.
Cranial circ. at 60 months (cm)	66	51.3 ± 0.2	51.4 ± 0.3	n.s.

Continuous data are reported as mean ± sem. Abbreviations: n.s. = not significant; BMI = body mass index; IQ = intelligence quotient; RPM = Raven’s progressive matrices; PI = ponderal index; circ. = circumference.

**Table 2:** Associations between exclusive breastfeeding and cognitive scores at five years.

GDMS Scale	Analysis	Total Population B (95% C.I.)	Girls B (95% C.I.)	Boys B (95% C.I.)
Locomotor	Univariate	0.3 (−3.6, 4.3)	1.5 (−3.9, 6.9)	−0.6 (−6.6, 5.7)
	Adjusted *	1.2 (−4.0, 6.5)	3.5 (−3.6, 10,6)	−1.9 (−11.0, 7.2)
Personal−social	Univariate	0.2 (−4.7, 5.1)	3.1 (−3.1, 9.5)	−2.3 (−10.1, 5.5)
	Adjusted *	2.9 (−3.5, 9.3)	6.2 (−2.1, 14.7)	−2.1 (−12.7, 8.5)
Hearing-language	Univariate	4.3 (−0.9, 9.6)	10.5 (2.4, 18.7) **	−0.47 (−7.7, 6.8)
	Adjusted *	4.3 (−2.4, 11.0)	11.0 (0.9, 21.2) **	−3.0 (−12.1, 6.1)
Eye-hand coordination	Univariate	−2.7 (−8.2, 2.3)	1.9 (−7.0, 10.9)	−6.0 (−13.0, 1.0)
	Adjusted *	−2.7 (−9.7, 4.3)	0.8 (−11.5, 13.2)	−6.2 (−16.4, 3.9)
Performance	Univariate	−1.3 (−5.1, 2.5)	−2.2 (−7.9, 3.4)	−0.1 (−5.6, 5.5)
	Adjusted *	−0.8 (−5.8, 4.1)	−2.7 (−10.5, 5.1)	0.7 (−7.4, 8.9)
Practical reasoning	Univariate	−1.6 (−6.6, 3,4)	0.8 (−8.2, 9.8)	−3.4 (−9.5, 2.7)
	Adjusted *	1.0 (−5.0, 7.0)	4.2 (−6.9, 15.2)	−4.0 (−11.0, 2.9)

GDMS = Griffiths Mental Development Scales; * Adjustment was made for maternal IQ, parents’ age, maternal pregravidic BMI, infants’ weight gain between 3 and 6 months and sex for the total population; ** *p* < 0.05.
